# ΔNp63α suppresses cells invasion by downregulating PKCγ/Rac1 signaling through miR-320a

**DOI:** 10.1038/s41419-019-1921-6

**Published:** 2019-09-12

**Authors:** Amjad A. Aljagthmi, Natasha T. Hill, Mariana Cooke, Marcelo G. Kazanietz, Martín C. Abba, Weiwen Long, Madhavi P. Kadakia

**Affiliations:** 1 Department of Biochemistry and Molecular Biology, Boonshoft School of Medicine, Wright State University, 3640 Colonel Glenn Highway, Dayton, OH 45435 USA; 2 Department of Systems Pharmacology and Translational Therapeutics, Perelman School of Medicine, University of Pennsylvania, Philadelphia, PA 19104 USA; 3 Centro de Investigaciones Inmunológicas Básicas y Aplicadas, Universidad Nacional de La Plata, CP1900 La Plata, Argentina

**Keywords:** Oncogenes, Cell signalling

## Abstract

ΔNp63α, a member of the p53 family of transcription factors, is overexpressed in a number of cancers and plays a role in proliferation, differentiation, migration, and invasion. ΔNp63α has been shown to regulate several microRNAs that are involved in development and cancer. We identified miRNA miR-320a as a positively regulated target of ΔNp63α. Previous studies have shown that miR-320a is downregulated in colorectal cancer and targets the small GTPase Rac1, leading to a reduction in noncanonical WNT signaling and EMT, thereby inhibiting tumor metastasis and invasion. We showed that miR-320a is a direct target of ΔNp63α. Knockdown of ΔNp63α in HaCaT and A431 cells downregulates miR-320a levels and leads to a corresponding elevation in PKCγ transcript and protein levels. Rac1 phosphorylation at Ser71 was increased in the absence of ΔNp63α, whereas overexpression of ΔNp63α reversed S71 phosphorylation of Rac1. Moreover, increased PKCγ levels, Rac1 phosphorylation and cell invasion observed upon knockdown of ΔNp63α was reversed by either overexpressing miR-320a mimic or Rac1 silencing. Finally, silencing PKCγ or treatment with the PKC inhibitor Gö6976 reversed increased Rac1 phosphorylation and cell invasion observed upon silencing ΔNp63α. Taken together, our data suggest that ΔNp63α positively regulates miR-320a, thereby inhibiting PKCγ expression, Rac1 phosphorylation, and cancer invasion.

## Introduction

ΔNp63α is a homolog of the p53 tumor suppressor gene and the dominant p63 isoform expressed in the proliferative basal layer of epithelial tissues^1–3^. Overexpression of ΔNp63α is frequently observed in squamous cell carcinoma (SCC) and basal cell carcinoma (BCC) where it has been shown to inhibit apoptosis and differentiation while promoting cell proliferation, thereby characterizing ΔNp63α as a proto-oncogene^4,5^. ΔNp63α has also been shown to inhibit EMT by downregulating genes that promote cell motility and mesenchymal phenotypes such as ZEB1 and Snail, thus inhibiting cell invasion^5–8^. Loss of ΔNp63α has been linked to increased invasiveness in SCC^9^. The contribution of ΔNp63α to cell invasion and metastasis appears to vary by cell type, as ΔNp63α has also been shown to increase basal-like breast cancer cell migration and invasion^10^. An improved understanding of ΔNp63α-mediated regulation of cell invasion will provide potential therapeutic inroads.

p63 exists as six different isoforms arising from alternative promoter usage and differential 3′ splicing. Transcription initiation from promoter 1 (P1) yields the TAp63 isoforms with a full N-terminal activation domain, while initiation from promoter 2 (P2) yields the ΔNp63 isoforms that have a truncated N-terminal domain. Moreover, alternative 3′ splicing of TAp63 and ΔNp63 leads to *α*, *β*, and *γ* isoforms^11,12^. While TAp63α and ΔNp63α generally have opposing functions in vivo, they both suppress tumor cell invasiveness^13,14^. ΔNp63α is of particular interest in skin cancer because it serves as a broad regulator of microRNA (miRNA) expression, including many that inhibit cell invasion^5,8,15–17^. miRNAs are small noncoding RNA molecules of 18–24 nucleotides in length. They regulate gene expression post-transcriptionally by binding to complementary sequences in the 3′-untranslated region (UTR) of their target mRNA, leading to translation inhibition or mRNA degradation^18,19^. Of particular relevance, miR-320a was previously shown to suppress colorectal cancer progression by directly binding to the 3′-UTR of the Rac1 mRNA, leading to downregulation of Rac1 protein levels^20^.

Rac1 belongs to the Rho family of small GTPases and plays fundamental roles in cellular proliferation, adhesion, invasion, migration, and gene transcription. Altered Rac1 expression and activity are frequently observed in human cancer^21,22^. Rac1 cycles between its active form (GTP-bound) and inactive form (GDP-bound) by the action of guanine nucleotide exchange factors that promote GTP loading and GTPase activating proteins (GAPs) that accelerate GTP hydrolysis^21^. Plasma membrane-associated active Rac1 induces actin polymerization at the edge of the cell, leading to formation of lamellipodia and promoting cell motility^23^. Importantly, Rac1 localization to the plasma membrane, binding to its effector proteins, and downstream signaling are also regulated via phosphorylation by a number of kinases^24–27^, although the specific nature of these post-translational events remains poorly understood. Rac1 activity is also regulated by protein kinase C (PKC), a family of phospholipid-dependent Ser/Thr kinases widely implicated in the control of cell proliferation, invasion, migration, and anticancer drug resistance^28–31^. In the last years, several studies have linked PKC to the activation of Rac1 and cancer cell motility^30–33^.

In this study, we identified miR-320a as a direct target of ΔNp63α. We demonstrated that ΔNp63α positively regulates miR-320a which in turn targets PKCγ 3′UTR, and thereby suppresses cell invasion. We showed that ΔNp63α downregulates PKCγ expression and Rac1 phosphorylation through miR-320a, thus suggesting a potentially novel mechanistic link between p63 and cancer invasiveness through the regulation of the Rac1 small GTPase.

## Results

### ΔNp63α induces miR-320a expression

**Fig. 1:**
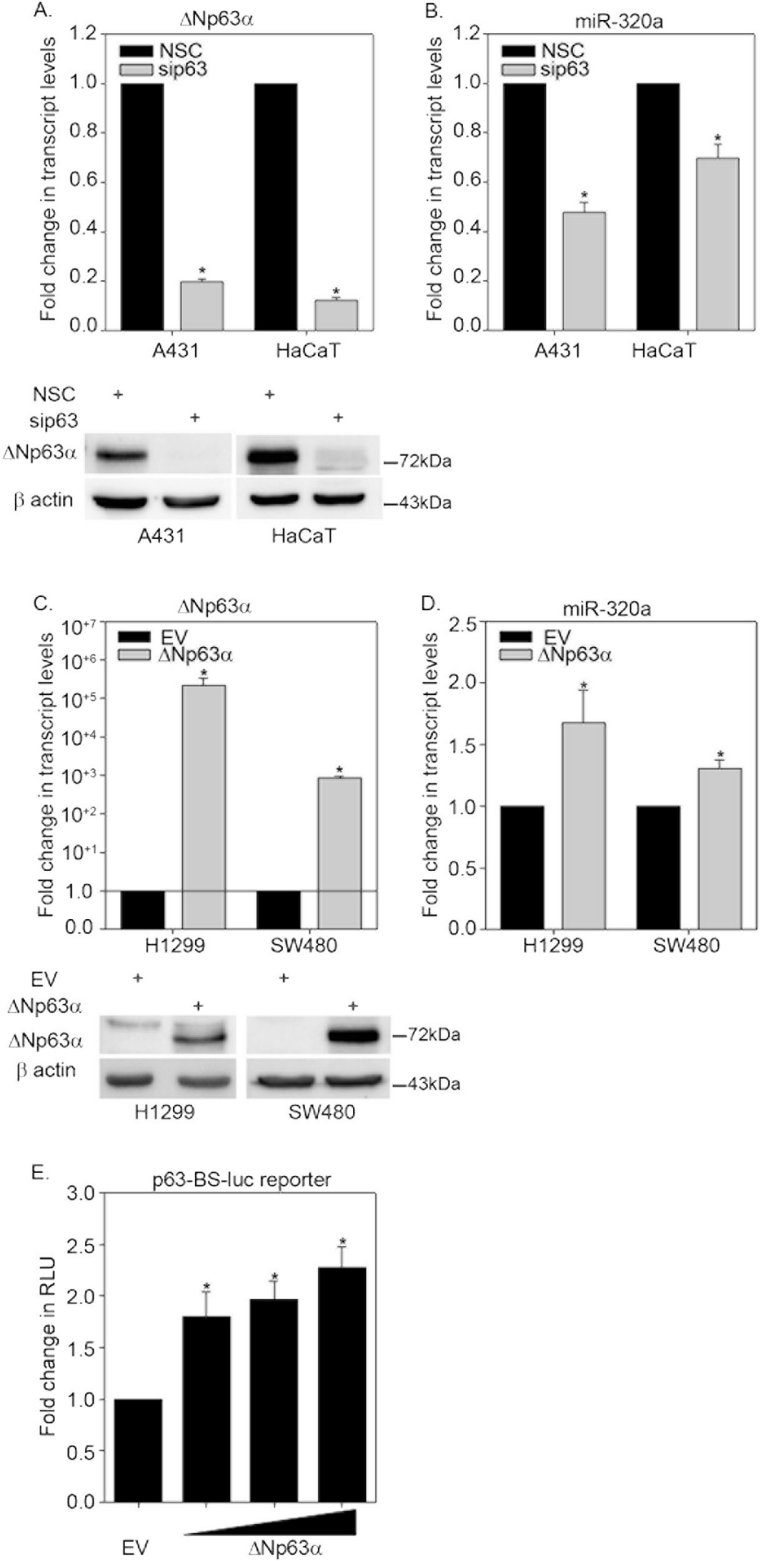
ΔNp63α positively regulates miR-320a. **a** A431 and HaCaT cells were transfected with non-silencing control siRNA (NSC) or siRNA specific to p63. Total RNA was extracted and ΔNp63α transcript level was measured by TaqMan based qRT-PCR. *y*-Axis represents the fold change in p63 transcript levels relative to NSC-transfected cells. Immunoblots of p63 in A431 and HaCaT cells transfected are shown in the bottom panels. **b** TaqMan based qRT-PCR was used to quantify miR-320a levels from the experiment described in (**a**). **c** H1299 and SW480 cells null for p63 were transfected with empty vector (EV) control or expression plasmid encoding ΔNp63α. Transcripts were quantified by qRT-PCR (upper panel) while protein levels were confirmed using immunoblot analyses (lower panel). **d** Taqman based qRT-PCR was used to quantify miR-320a levels from the experiment described in (**c**). Immunoblot with β-actin was performed to confirm equivalent protein loading. Error bars represent standard deviation. Significant changes (*P* ≤ 0.05) relative to controls are indicated with an asterisk. **e** H1299 cells were co-transfected with p63-BS-Luc reporter construct along with either empty vector or increasing concentrations of ΔNp63α as indicated. Cells were subjected to dual luciferase assay at 24 h after transfection. The *y*-axis represents fold change in relative luciferase units (RLU) compared with cells transfected with empty vector. RLU values are shown as means ± S.E.M. from *n* = 3 experiments

miR-320a functions as a tumor suppressor in glioma, breast and colorectal cancers by suppressing cell migration, invasion, and proliferation^20,34–36^. To determine if ΔNp63α regulates miR-320 levels, we either knocked down p63 in HaCaT cells and A431 cells, which predominantly express the ΔNp63α isoform of p63^14^, or overexpressed ΔNp63α in p63 null SW480 and H1299 cells. Both p63 knockdown and overexpression were confirmed by Western blot and quantitative reverse transcription polymerase chain reaction (qRT-PCR) (Fig. 1a, c). We observed that p63 knockdown led to a decrease in miR-320a transcript levels (Fig. 1b) whereas overexpression of ΔNp63α, led to an increase in the miR-320a levels (Fig. 1d).

A previous p63 ChIP-seq study (GSE59827), demonstrated strong p63 binding site 6 kb (chr8:22,239,143–22,239,162) downstream of miR-320a gene^37,38^. To confirm that miR-320a is a direct target of ΔNp63α, we cloned this region containing the p63 binding site into the pGL3-promoter luciferase vector (p63-BS-luc reporter). Next, we co-transfected H1299 cells with p63-BS-luc reporter along with increasing concentrations of the expression plasmid encoding ΔNp63α. Co-transfection of ΔNp63α led to a dose-dependent increase in the luciferase activity of p63-BS-luc reporter (Fig. 1e). Taken together, these results demonstrate that ΔNp63α directly regulates miR-320a transcription levels.

### ΔNp63α negatively regulates phosphorylation of Rac1 at Ser71

**Fig. 2:**
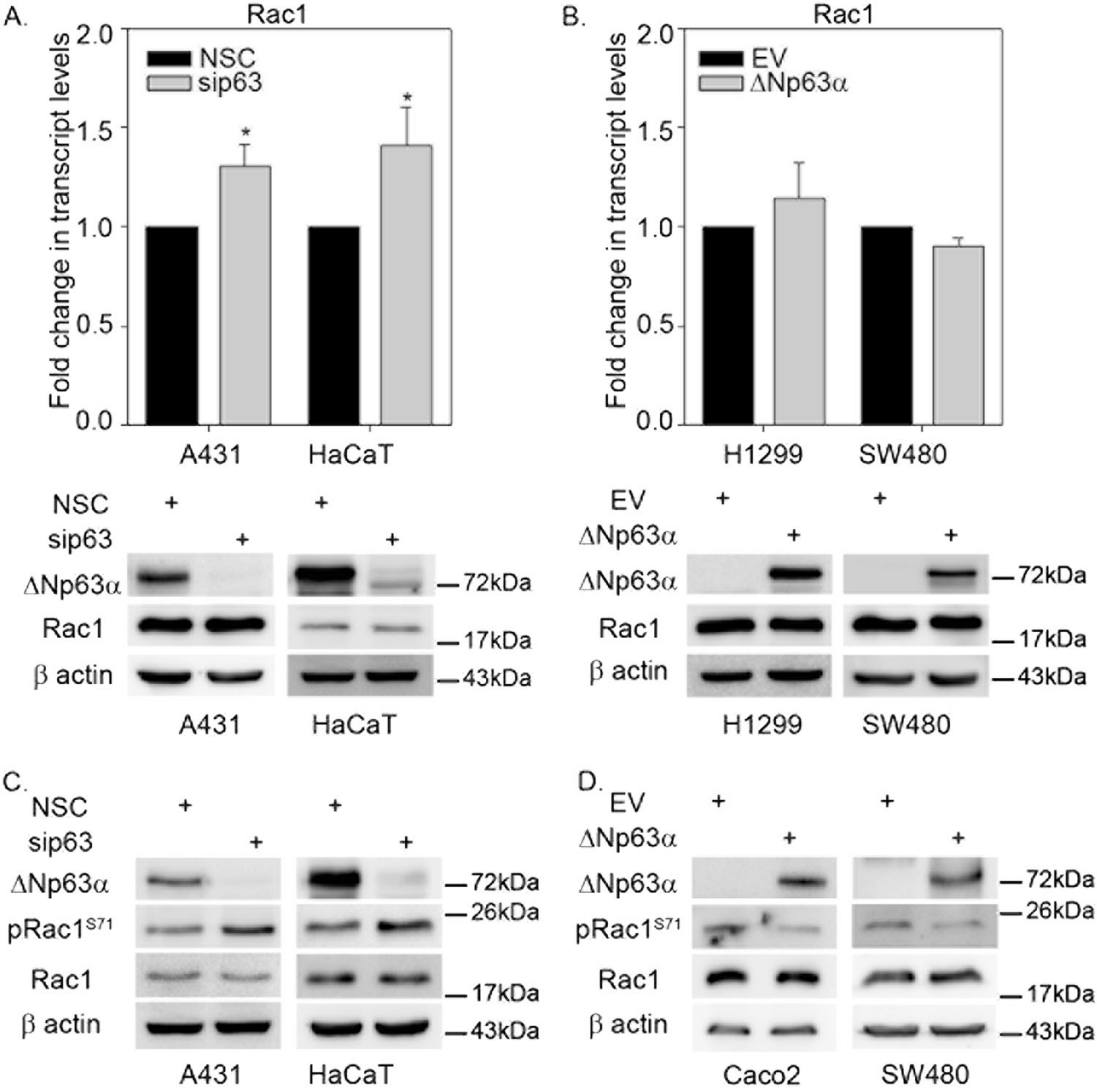
ΔNp63α negatively regulates Rac1 phosphorylation. **a** A431 and HaCaT cells were transfected with non-silencing control siRNA (NSC) or siRNA targeting p63. **b** H1299 and SW480 cells were transfected with empty vector (EV) control or expression plasmid encoding ΔNp63α. The change in transcript and protein levels of Rac1 were measured by TaqMan based qRT-PCR (* indicates *P* ≤ 0.05) and immunoblot analysis, respectively. Error bars represent standard deviation. **c** A431 and HaCaT cells were transfected with nonsilencing control siRNA (NSC) or siRNA against p63. **d** Caco2 and SW480 cells were transfected with empty vector control or ΔNp63α and subjected to immunoblot analysis for the indicated proteins. The change in Rac1 protein expression was measured by immunoblot analysis using Rac1 and pRac1 (S71) antibodies as indicated. Immunoblot with β-actin was performed to confirm equivalent protein loading

miR-320a has been shown to suppress colorectal cancer progression by targeting Rac1^20^. Since we observed that ΔNp63α positively regulates miR-320a, next we wanted to determine whether ΔNp63α negatively regulates Rac1-expression levels. Although ΔNp63α knockdown in both HaCaT and A431 cells led to a modest increase in Rac1 transcript levels, the total Rac1 protein levels remained unchanged (Fig. 2a). Consistently, ΔNp63α overexpression in ΔNp63α-deficient H1299 and SW480 cells, also did not affect Rac1 transcript and protein levels (Fig. 2b). Together these data indicate that although ΔNp63α positively regulates miR-320a, it does not inhibit Rac1 protein levels.

Notably, despite the lack of changes in total Rac1 upon modulation of ΔNp63α expression, Western blot analysis revealed elevated immunoreactivity using a phospho-Ser71 antibody, suggesting that the Rac1 phosphorylation status could be regulated by ΔNp63α expression. Immunoblot analysis indicated that the knockdown of ΔNp63α in A431 and HaCaT cells led to an increase in pRac1 (S71) levels (Fig. 2c). Conversely, ectopic expression of ΔNp63α in Caco2 and SW480 cells resulted in a decrease in pRac1 levels, with no change in total Rac1 expression (Fig. 2d). Taken together, these results demonstrate that ΔNp63α negatively regulates Rac1 phosphorylation without affecting total Rac1 levels.

### Increased invasion observed with loss of ΔNp63α is Rac1-dependent

**Fig. 3:**
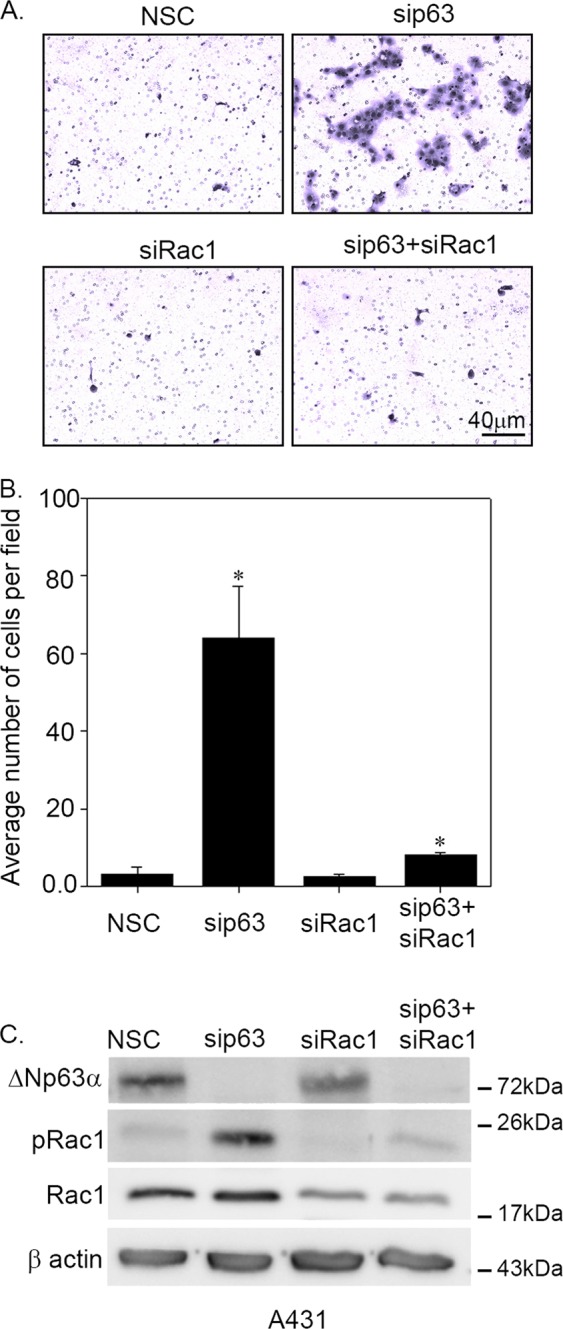
Knockdown Rac1 reversed increased cell invasion observed upon ΔNp63α knockdown. A431 cells were transfected with NSC siRNA, p63 siRNA, and/or Rac1 siRNA as indicated. At 24 h after the second round of transfection, 8.0 × 10^4^ cells were subjected to Matrigel-based invasion assay (**a**) and the number of invading cells was quantitated after 21 h (**b**). The *y*-axis indicates the average number of cells invaded per field. Error bars represent standard deviation. Significant changes (*P* ≤ 0.05) relative to NSC controls are indicated with an asterisk. **c** The change in protein expression was measured by immunoblot analysis using Rac1 and pRac1 (S71) antibodies to measure unphosphorylated and phosphorylated levels of Rac1, respectively. Immunoblot with β-actin was performed to confirm equivalent protein loading

We next examined whether ΔNp63α regulates cell invasion in a Rac1-dependent manner. The effects of ΔNp63α, Rac1, or combined ΔNp63α/ Rac1 silencing on cell invasion in A431 cells were assessed using a Matrigel-based transwell assay. As previously reported^14^, ΔNp63α knockdown significantly increased the number of invading cells (Fig. 3a). Rac1 knockdown alone decreased cell invasion when compared to control cells transfected with nonsilencing control (NSC). Knockdown of both ΔNp63α and Rac1 reversed the increased cell invasion observed upon ΔNp63α knockdown alone (Fig. 3a, b). ΔNp63α and Rac1 knockdown was confirmed by immunoblot analysis in this cell line (Fig. 3c). As expected, silencing Rac1 significantly decreased pRac1 levels observed with ΔNp63α siRNA depletion. Thus, these results strongly suggest that ΔNp63α inhibits the cell invasion via Rac1 phosphorylation.

### miR-320a counteracts the effect of ΔNp63α silencing on Rac1 phosphorylation and cell invasion

**Fig. 4:**
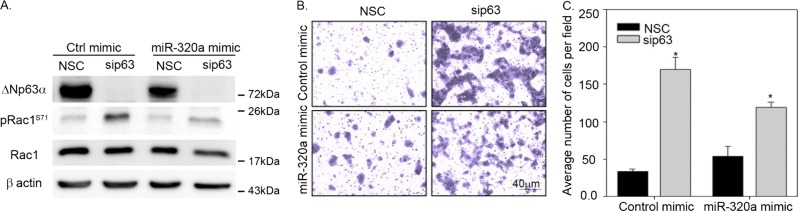
Overexpression of a miR-320a mimic rescues the effect of ΔNp63α knockdown on Rac1 phosphorylation and cell invasion. A431 cells were transfected with either non-silencing control (NSC) or sip63 in conjunction with a negative control mimic or miR-320a mimic for two rounds of transfections. **a** The change in indicated protein levels were analyzed via immunoblotting with p63, Rac1, and pRac1 (S71) antibodies as indicated. Immunoblot with β-actin was performed to confirm equivalent protein loading. Twenty four hour after the second of transfection, 8.0 × 10^4^ cells were subjected to Matrigel-based invasion assay (**b**) and the number of invading cells was quantitated after 21 h (**c**). The *y*-axis indicates the average number of cells invaded per field +1 standard deviation. Significant changes (*P* ≤ 0.05) relative to cells transfected with NSC and control mimic are indicated with an asterisk

We next examined whether the negative regulation of Rac1 phosphorylation by ΔNp63α is dependent on miR-320a. To test this, we transfected A431 cells with siRNA against p63 or NSC in combination with miR-320a mimic or its negative mimic control. ΔNp63α knockdown led to an increase in pRac1 levels, as expected. Co-expression of miR-320a mimic reversed the increased pRac1 levels observed upon ΔNp63α knockdown to basal levels (Fig. 4a). Similar results were observed in HaCaT cells (Supplementary Fig. 1A). These results clearly demonstrate that miR-320a mimic counteracts the effect of ΔNp63α knockdown on pRac1 levels.

To further determine whether miR-320a mimic can also rescue the increased cell invasion observed upon p63 knockdown, A431 cells were transfected with control mimic or miR-320a mimic, either in presence or absence of p63 siRNA, and assessed for cell invasion. Overexpression of the miR-320a mimic in NSC control cells had only a minimal effect on cell invasion, consistent with baseline ΔNp63α-induced miR-320a levels in A431 cells. As expected, knockdown of ΔNp63α significantly increased the number of invading cells (Fig. 4b, c). Overexpression of the miR-320a mimic in cells transfected with siRNA to p63 showed a dramatic decrease in the number of invading cells when compared to cells transfected with siRNA to p63 alone (Fig. 4b, c). Similar results were observed in HaCaT cells (Supplementary Fig. 1B). Taken together, these results clearly indicate that miR-320a mimic reversed increased cell invasion observed upon ΔNp63α knockdown by targeting Rac1 phosphorylation.

### PKCγ expression levels are negatively regulated by ΔNp63α and miR-320a

**Fig. 5:**
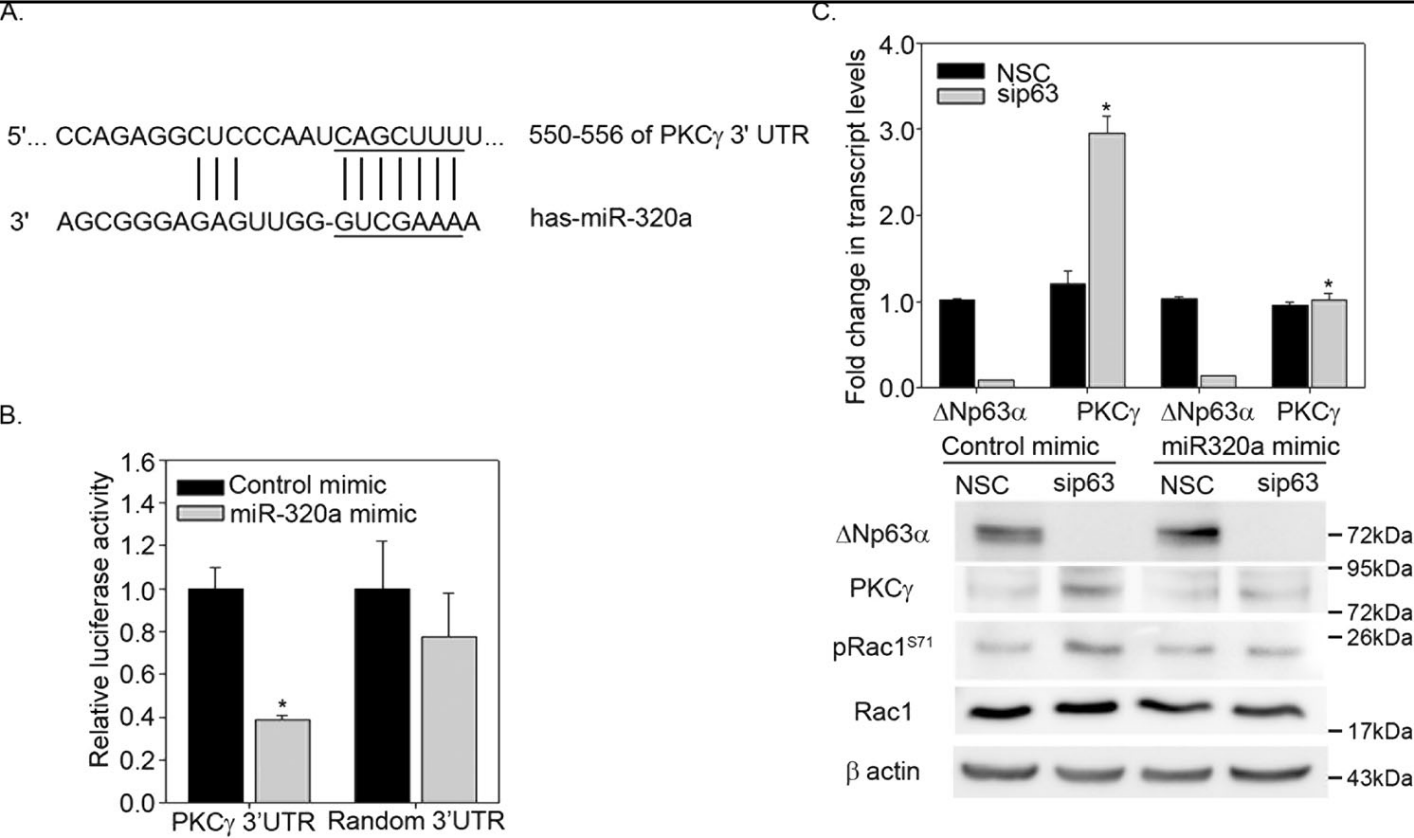
Negative regulation of PKCγ expression by ΔNp63α is dependent on miR-320a. **a** A putative miR-320a binding site in PKCγ 3′UTR identified by Target Scan. **b** A431 cells were co-transfected with a luciferase reporter carrying PKCγ 3′UTR or a random 3′UTR along with control mimic or miR-320a mimic. At 24 h post transfection, a luciferase reporter assays were performed. *y*-Axis represents the relative change in the luciferase activity. Significant changes (*P* ≤ 0.05) relative to control mimic are indicated with an asterisk. **c** HaCaT cells were transfected with either NSC siRNA or p63 siRNA in conjunction with a negative control mimic or miR-320a mimic as indicated. Total RNA was extracted and transcript levels of ΔNp63α and PKCγ was measured by Taqman based qRT-PCR (upper panel). *y*-Axis represents the fold change in ΔNp63α and PKCγ transcript levels relative to NSC-transfected cells. Error bars indicate standard deviation. Significant changes (*P* ≤ 0.05) relative to respective NSC controls are indicated with an asterisk. The change in indicated protein levels were measured analyzed via immunoblotting with p63, PKCγ, Rac1, and pRac1 (S71) antibodies as indicated (lower panel). Immunoblot with β-actin was performed to confirm equivalent protein loading

Next, we wished to elucidate the mechanism by which the ΔNp63α/miR-320a axis negatively regulates Rac1 phosphorylation. Therefore, we examined whether miR-320a negatively regulates pRac-1 levels by targeting a kinase. Target analysis using miRDB (www.mirdb.org) and TargetScan (www.targetscan.org)^39,40^ both identified PKCγ as a predicted target of miR-320a (Fig. 5a). To validate that PKCγ is a target for miR-320a, we co-transfected A431 cells with a luciferase reporter construct that contains the PKCγ 3′UTR or a random control 3′UTR with either miR-320a mimic or a negative control mimic. We observed that miR-320a mimic led to a significant reduction in the PKCγ 3′UTR luciferase activity when compared to the co-transfection with the negative control mimic (Fig. 5b). No significant change in the luciferase activity was observed when the random 3′UTR luciferase reporter was co-transfected with miR-320a mimic. This confirms that PKCγ is a target for miR-320a.

Next, we wanted to determine if ΔNp63α/miR-320a axis regulates PKCγ expression, HaCaT cells were transfected with either NSC or ΔNp63α siRNA, along with miR-320a mimic or negative mimic control (Fig. 5c). We observed that ΔNp63α knockdown led to a significant upregulation in PKCγ transcript and protein levels, with a concomitant increase in pRac1 levels (Fig. 5c). Overexpression of miR-320a mimic reduced PKCγ transcript and protein to basal levels, thereby reversing the effects of p63 silencing (Fig. 5c). Taken together, these results demonstrate that ΔNp63α negatively regulates PKCγ expression through upregulation of miR-320a.

### Silencing PKCγ reverses the increased pRac1 levels and cell invasion observed upon loss of ΔNp63α

**Fig. 6:**
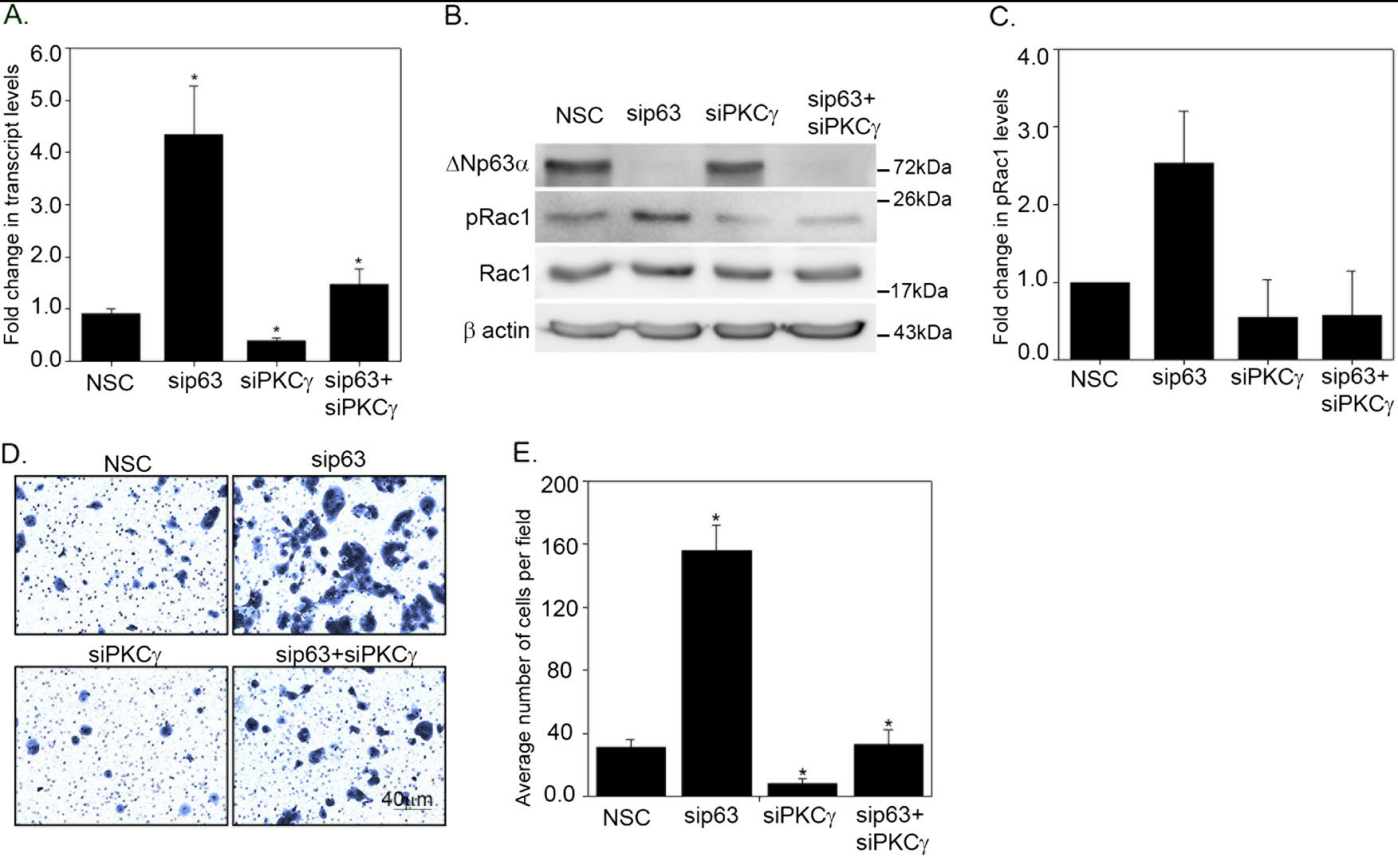
PKCγ knockdown reversed the effect of ΔNp63α knockdown on pRac1 and cell invasion. A431 cells were transfected with either non-silencing control (NSC), sip63 alone, siPKCγ alone, or sip63 in conjugation with siPKCγ for two rounds of transfections. **a** At 24 h post transfection, the change in transcript levels of PKCγ was measured by TaqMan based qRT-PCR. *y*-Axis represents the fold change in PKCγ transcript levels relative to NSC-transfected cells. Error bars represent standard deviation. **b** The change in indicated protein levels were analyzed via immunoblotting with p63, Rac1 and pRac1 (S71) antibodies as indicated. Immunoblot with β-actin was performed to confirm equivalent protein loading. **c** Quantification of pRac1 levels. Relative protein values are shown as means ± S.E.M. from *n* = 3 experiments. Twenty four hour after the second round of transfection, 8.0 × 104 cells were subjected to Matrigel-based invasion assay (**d**) and the number of invading cells was quantitated after 21 h (**e**). The *y*-axis indicates the average number of cells invaded per field +1 standard deviation. Significant changes (*P* ≤ 0.05) relative to NSC controls are indicated with an asterisk

We next examined whether ΔNp63α-mediated inhibition of Rac1 phosphorylation and cell invasion is mediated by miR-320a-PKCγ signaling. To this end, we investigated the effects of silencing p63 and/or PKCγ on pRac1 levels and cell invasion. p63 knockdown alone led to a significant increase in PKCγ transcript levels (Fig. 6a), whereas knockdown of PKCγ alone led to a further reduction in PKCγ transcript levels when compared to cells transfected with nonsilencing control. Knockdown of both p63 and PKCγ led to a reduction in the increased PKCγ transcript levels observed upon knockdown of p63 alone (Fig. 6a). Interestingly, knockdown of both p63 and PKCγ reversed the increased pRac1 levels observed upon p63 knockdown (Fig. 6b, c).

Next, we examined the effects of p63 and/or PKCγ silencing on cell invasion. As expected, p63 knockdown alone led to increased cell invasion (consistent with Fig. 4b). Knockdown of both p63 and PKCγ led to a reduction in cell invasion comparable to control cells transfected with NSC (Fig. 6d, e). Taken together, these results suggest that the inhibition in Rac1 phosphorylation and cell invasion by ΔNp63α and miR-320a is through downregulating PKCγ.

### PKC inhibitor mimics the effect of ΔNp63α on Rac1 phosphorylation and invasion

**Fig. 7:**
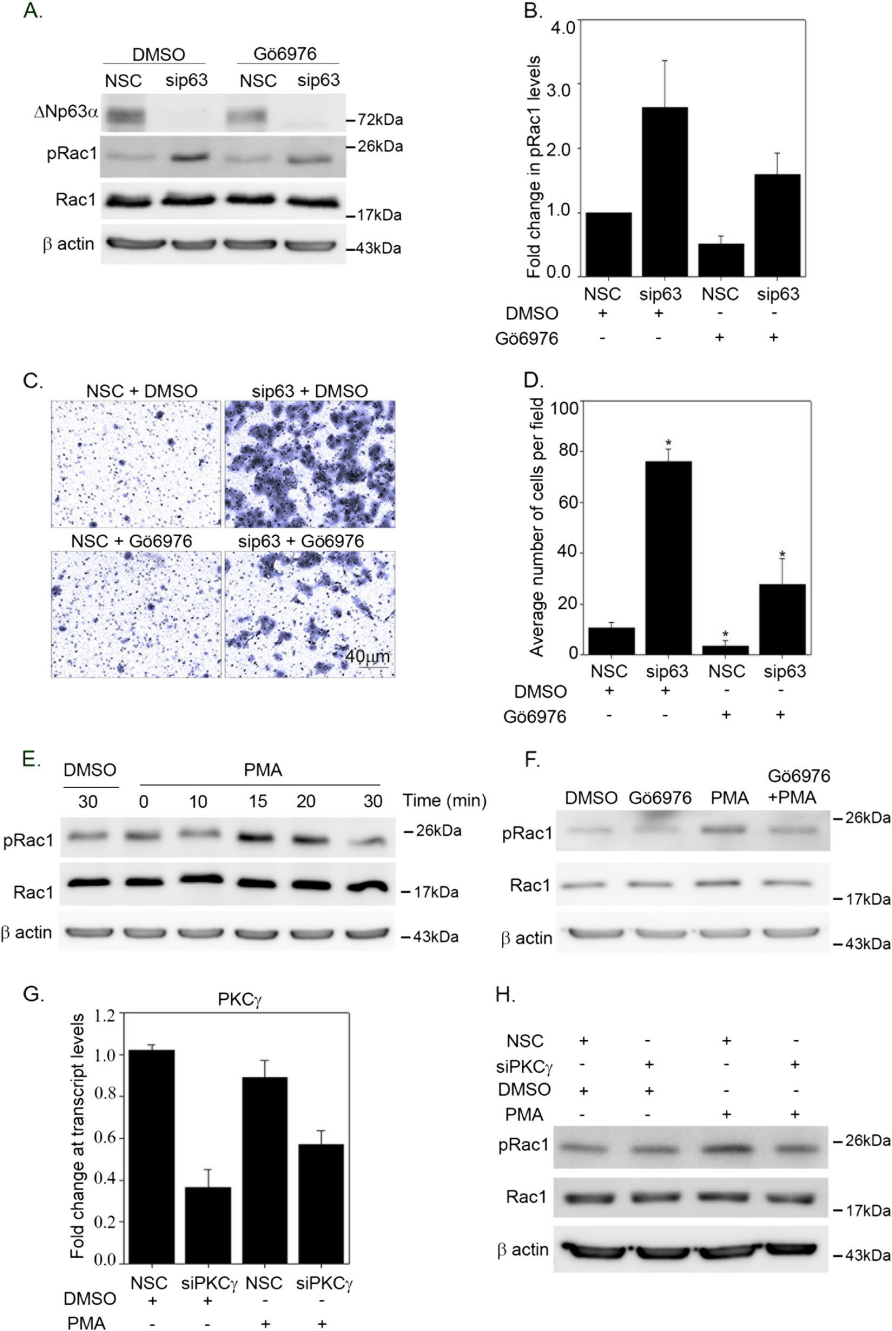
ΔNp63α inhibits Rac1 phosphorylation and invasion by reducing PKCγ levels. A431 cells were transfected with either NSC siRNA or siRNA targeting p63 for two rounds of transfections followed by treatment with DMSO or Gӧ6976 for 2 h as indicated. **a** The change in indicated protein levels were analyzed via immunoblotting with p63, Rac1, and pRac1 (S71) antibodies as indicated. Immunoblot with β-actin was performed to confirm equivalent protein loading. **b** The fold change in pRac1 levels relative to NSC DMSO-treated cells levels. Relative protein values are shown as means ± S.E.M. from *n* = 3 experiments. **c** At 24 h after the second round of transfection, 8.0 × 10^4^ cells were subjected to Matrigel-based invasion assay (**c**) and the number of invading cells was quantitated after 21 h. **d** The *y*-axis indicates the average number of cells invaded per field +1 standard deviation. Significant changes (*P* ≤ 0.05) relative to NSC controls are indicated with an asterisk. **e** A431 cells were incubated with DMSO or 100 nM of PMA for the indicated times. The change in indicated protein levels were analyzed via immunoblotting with Rac1 and pRac1 (S71) antibodies as indicated. **f** A431 cells were treated with DMSO or Gö6976 for 2 h and followed by incubation with 100 nM of PMA for 15 min. The change in indicated protein levels were analyzed via immunoblotting as indicated. **g** A431 cells were transfected with nonsilencing control siRNA (NSC) or siRNA specific to PKCγ followed by treatment with DMSO or 100 nM PMA for 15 min. Total RNA was extracted and transcript levels of PKCγ was analyzed by qRT-PCR. *y*-Axis represents the fold change in PKCγ transcript levels relative to NSC-transfected cells. The change in Rac1 and pRac1 levels was measured by immunoblot analysis (**h**). Immunoblot with β-actin was performed to confirm equivalent protein loading

To further confirm that the increase in pRac1 levels upon p63 knockdown is due to PKCγ upregulation, we first tested the effect of Gö6976, an inhibitor of conventional PKC*α*, *β* and *γ* isoforms, on Rac1 phosphorylation in cells transfected with nonsilencing control or siRNA to p63. Consistent with PKCγ knockdown experiments (Fig. 6), we observed that the increase in pRac1 levels observed upon p63 knockdown was reversed by treatment with Gö6976 (Fig. 7a, b). Next, we examined the effect of PKCγ inhibition on cell invasion in cells transfected with NSC or siRNA to p63. Increased cell invasion observed upon p63 knockdown was significantly reduced when cells were treated with Gö6976 (Fig. 7c, d). As a control, we also examined the effect of p63 silencing on PKCα, a ubiquitously expressed PKC that is also inhibited by Gö6976. Our results revealed that p63 knockdown (Supplementary Fig. 2a) did not significantly induce PKCα either at the transcript or protein level (Supplementary Fig. 2b, d). These results clearly indicate that ΔNp63α inhibition of Rac1 phosphorylation is mediated through a reduction of PKCγ levels.

Next, we investigated the effect of the PKC activator phorbol 12-myristate 13-acetate (PMA) on Rac1 phosphorylation. A431 cells were treated with either 100 nM PMA or vehicle for various time intervals (0–30 min). PMA treatment led to an increase in pRac1 levels that peaked at 15 min, followed by a gradual decrease at 20 and 30 min (Fig. 7e). This effect was inhibited by Gö6976 treatment (Fig. 7f). To further confirm that the increase in pRac1 levels involves PKCγ activation, we examined the effect of silencing PKCγ and treating with PMA on pRac1 levels. Silencing PKCγ abolished the increase in pRac1 induced by PMA (compare PMA/siPKCγ with PMA/NSC in Fig. 7g, h). Taken together, these results clearly confirm that Rac1 phosphorylation is induced by PKCγ, and that ΔNp63α and miR-320a inhibit Rac1 phosphorylation and hence cell invasion via targeting PKCγ.

### Inverse correlation observed between ΔNp63α, miR-320a expression when compared to PKCγ expression in human cancers

**Fig. 8:**
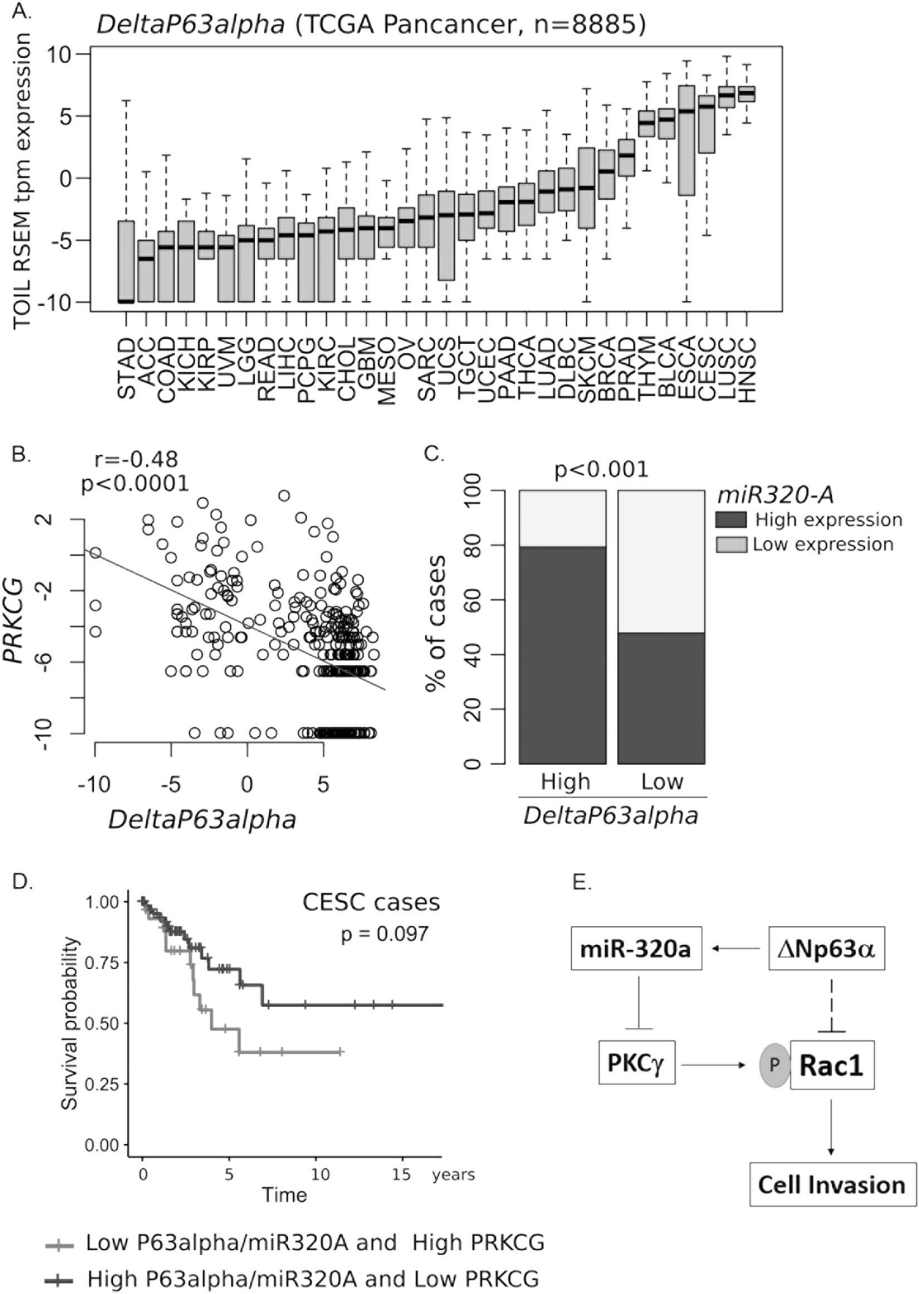
Gene-expression analysis of ΔNp63α and miR-320a in correlation with PKCγ expression among human cancers. **a** TCGA Pan-Cancer dataset were used to analyze ΔNp63α expression in 8885 primary cancer cases from 32 tumor locations. **b** Correlation of ΔNp63α and PKCγ levels within the cervical squamous cell carcinomas (CESC) dataset. **c** Expression of ΔNp63α and PKCγ in 296 CESC samples divided into high and low categories for each mRNA using StepMiner algorithm. **d** Kaplan–Meier curve and log-rank test for the survival of 100 CESC patients categorized as either low ΔNp63α or miR-320a with high PKCγ (*n* = 32) or high ΔNp63α and miR-320a with low PKCγ (*n* = 68). **e** Model representing the regulation of Rac1 phopshorylation by ΔNp63α via the miR-320a/PKCγ axis

To examine the potential clinical relevance of PKCγ downregulation by ΔNp63α/miR-320a axis, we first analyzed the level of ΔNp63α expression among 32 different cancer types using TCGA Pan-Cancer datasets (Fig. 8a). We found the highest expression of ΔNp63α in cervical, lung and head and neck SCC. In silico analysis revealed a significant negative correlation between ΔNp63α and PKCγ expressions (Fig. 8b). A significant correlation was found between the expressions of ΔNp63α and miR-320a (*p* < 0.0001, Fisher’s exact test) with 80% of cervical squamous cell carcinomas (CESC) tumors expressing high levels of ΔNp63α also showing high levels of miR-320a (Fig. 8c). Finally, Kaplan–Meier analysis carried out in CESC revealed that patients with low p63/miR-320a levels and high-PKCγ levels have poor survival when compared with patients with high p63/miR-320a levels and PKCγ levels (Fig. 8d).

## Discussion

ΔNp63α, a master regulator of epithelial differentiation, has been shown to suppress cell invasion through downregulation of both mRNAs and microRNAs involved in cell adhesion and motility, however, a detailed molecular understanding of ΔNp63α function in cell invasion is lacking^11,15–17^. Here, we have identified a novel signaling mechanism by which ΔNp63α positively regulates miR-320a to target PKCγ, resulting in suppression of Rac1 phosphorylation and thus cell invasion (Fig. 8e). Investigation into this novel mechanism could yield new therapeutic options in the treatment of metastatic cancers.

We have previously shown that increased expression of ΔNp63α increases proliferation and is a hallmark of nonmelanoma skin cancers such as BCC and squamous cell carcinoma^4,41,42^. Interestingly, while ΔNp63α canonically functions as an oncogene by increasing proliferation, it generally prevents tumor metastasis by suppressing invasiveness^4,8,14^. As a result, direct therapeutic targeting of ΔNp63α is undesirable as it would be expected to slow tumor growth while increasing invasiveness. Rather, targeting downstream effectors of ΔNp63α involved in cell invasion pathways such as the miR-320a/PKCγ/pRac1 axis identified in this study may have therapeutic value in slowing the progression of SCC. In addition, previous studies have clearly demonstrated that ΔNp63α plays an important role in maintenance and proliferation of epidermal stem cells and thereby normal keratinocyte homeostasis^43,44^. In addition, both p63 and Rac1 have been shown to be required for wound healing^14,45^. Since no studies to date have shown an association between miR-320a, PKCγ and pRac1 in normal epidermal homeostasis or wound healing, our findings on the ΔNp63α/miR320a/PKCγ/pRac1 axis are novel and suggest a role in both cancer cells as well as normal keratinocyte homeostasis and/or wound healing.

miR-320a, identified here as a novel ΔNp63α-regulated miRNA, is a known tumor suppressive miRNA that is downregulated in many metastatic cancers^20,36,46,47^. Loss of miR-320a leads to increased colon cancer cell proliferation and increased bladder carcinoma invasion^34,48^. Further, loss of miR-320a was shown to promote invasion and metastasis in tongue SCC and breast cancer^49,50^. Conversely, high expression of miR-320a would appear to be a good prognostic indicator in cancer, and the use of a miR-320a mimic in SCC therapy would be expected to have therapeutic value.

Our results further show that ΔNp63α positively regulates miR-320a, which results in reduced expression of its target PKCγ. This PKC, originally thought to be expressed only in neurons, has only recently been identified as a potential mediator of colon and breast cancer invasiveness^51,52^. PKCγ knockdown in the mouse periventricular nucleus has been shown to significantly reduce total GTP-bound Rac1 levels and the number of phospho-Rac1 positive neurons^53^. Increased PKCγ protein levels has been shown to promote the migration of colon cancer cells whereas a reduction in PKCγ levels induces cell adhesion and proliferation^52^. Moreover, the therapeutic potential of PKCγ inhibition has been hindered by the lack of an isoform specific inhibitor, but broad spectrum PKC inhibitors such as midostaurin have been shown to suppress tumorigenesis in AML and advanced systematic mastocytosis^54^.

We further show that ΔNp63α regulates the phosphorylation of Rac1 through the miR-320a-PKCγ axis. Although miR-320a was shown to affect the expression of Rac1 in colorectal cancer cells^20^, ΔNp63α did not change total Rac1 levels in four different cell lines used in our study, including SW480 colon cancer cells. Although the reasons behind these differences are not known, we have clearly shown that p63 negatively regulates Rac1 phosphorylation using pRac1 specific antibodies. The role of Rac1 in the acquisition of invasive and metastatic phenotypes and thus cancer progression has been well established, and increased expression of Rac1 has been associated with poor prognostic outcome for a number of cancers^55–57^. The effect of blocking Rac1 phosphorylation on invasion, migration and proliferation in SCC has not been explored. Thus, additional testing is needed to determine the functional consequences and therapeutic potential of perturbing the miR-320a/PKCγ/pRac1 pathway.

In summary, the work presented in this study elucidates one arm by which ΔNp63α functions to inhibit cell invasion. ΔNp63α regulation of miR-320a inhibits PKCγ/pRac1 signaling leading to inhibition of cell invasion. This study strongly links ΔNp63α to regulation of Rho small GTPases, and thus to a role in cytoskeleton rearrangement and subsequently cell motility as it pertains to invasion and migration of tumor cells.

## Materials and methods

### Cell culture and reagents

The SCC cell line A431, the human nonsmall cell lung carcinoma cell line H1299, and the colorectal adenocarcinoma SW480 and Caco2 cell lines were purchased from American Type Culture Collection (Manassas, VA, USA). The nontumorigenic immortalized human keratinocyte HaCaT cell line was obtained from Dr. Nancy Bigley (Wright State University). All cell lines were grown in Dulbecco’s modified Eagle’s medium (DMEM) supplemented with 8% fetal bovine serum (FBS) and 250 U penicillin and 250 μg streptomycin. The PKC inhibitor Gö6976 was obtained from Sigma-Aldrich (St. Louis, MO). PMA was purchased from Cell Signaling Technology (Danvers, MA).

### miRNA, siRNA, and DNA transfections

miR-320a mimic and miRNA mimic negative control were obtained from Dharmacon (Lafayette, CO, USA). A total of 40 nM of miR-320a mimic or mimic negative control was transfected into A431 or HaCaT cells using Lipofectamine RNAi-Max per manufacturer’s instructions (Life Technologies, Carlsband, CA, USA). Rac1, PKCγ, and p63 knockdown studies conducted in HaCaT and A431 cells were performed by two rounds of siRNA transfection using Lipofectamine RNAi-Max. Rac1 and p63 siRNAs used in this study were purchased from Qiagen (Valencia, CA, USA) and PKCγ siRNA was purchased from Dharmacon (Lafayette, CO, USA). The ΔNp63α expression plasmid or the empty pcDNA3.1 control plasmid were transiently transfected into H1299, SW480 or Caco2 cells using Lipofectamine 2000 (Invitrogen, Carlsbad, CA, USA) as reported previously^4^. Cells were harvested 24 h after transfection and cell pellets were used for immunoblot analysis and extraction of total RNA for qRT-PCR studies.

### Western blot analysis

Cells were lysed in a buffer containing 50 mM Tris-HCl pH 8, 120 mM NaCl, 5 mM sodium pyrophosphate phosphatase inhibitor (NaPPi), 10 mM NaF, 30 mM paranitrophenylphosphate, 1 mM benzamidine, 0.1% NP-40, 1% Triton X-100 and 0.2 mM PMSF, 100 nM sodium orthovanadate, and supplemented with 10% protease inhibitor cocktail (Sigma, St. Louis, MO). Immunoblot analysis was carried as described previously^14^. Proteins were detected using the following antibodies: rabbit polyclonal anti-p63 [N2C1] (Gene Tex, Irvine, CA, USA), mouse monoclonal anti-Rac1 [23A8] (Abcam, Cambridge, MA, USA), rabbit polyclonal anti-Rac1 (C-11), rabbit polyclonal anti-phospho-Rac1 (Ser71), mouse monoclonal anti-β-actin antibody (Santa Cruz Biotechnology, Santa Cruz, CA, USA), and rabbit polyclonal anti-PKCγ (ABclonal Science, Woburn, MA, USA). Appropriate horseradish peroxidase-conjugated secondary antibodies (Promega, Madison, WI, USA) were used for chemiluminescence detection with a Western Lightning Plus chemiluminescent kit (Perkin Elmer, Waltham, MA, USA).

### Cloning and Luciferase reporter assay

The fragment of miR-320a gene enhancer region containing p63 binding site (chr8:22,239,143–22,239,162)^37,38^ was PCR amplified using the following primers: forward primer [5′-CCGGTACCGTGTTGGAACTACAGGCATG-3′] and the reverse primer [5′-GCAACTCGAGCATGTAAGGGTCAAGGCGAT-3′]. Amplified fragment was subcloned into pGL3-promoter luciferase vector (pGL3-promoter-Luc, Promega) via KPN1 and XHO1 sites. The resulted construct is designated as p63-BS-luciferase reporter. H1299 cells were plated on 24-well plates and transfected with p63-BS-luciferase reporter and Renilla luciferase constructs to normalize for transfection efficiency along with either an empty vector control or expression plasmid encoding ΔNp63α. At 24 h post transfection, cells were harvested in passive lysis buffer and subjected to Dual luciferase assay as per manufacturer’s protocol (Promega, Madison, WI). The relative luciferase activity was calculated as the ratio of Firefly luciferase activity to Renilla luciferase activity and normalized to empty pLG3 promoter vector transfected with increasing concentration of ΔNp63α.

To determine whether PKCγ is a direct target of miR-320a, luciferase reporter constructs containing RenSP luciferase gene cloned upstream of either the PKCγ 3'UTR or a random control 3′UTR were obtained from SwitchGear Genomics (Carlsbad, CA, USA). A431 cells were co-transfected with either PKCγ 3′UTR or a random 3UTR along with 100 nM of control mimic or miR-320a mimic. At 24 h post transfection, cells were assayed for the luciferase activity using LightSwitch Luciferase Assay Kit (SwitchGear Genomics, Carlsbad, CA, USA).

### Cell invasion assay

Cell invasion was assessed using a two-chamber transwell system. A total of 8 × 10^4^ transiently transfected A431 or HaCaT cells were suspended in serum-free DMEM medium and seeded into 8 μm pore size inserts (BD Biosciences) that were coated with 1 mg/ml Matrigel (BD Biosciences) and placed into 24-well plate. Then, DMEM containing 8% FBS was added to the bottom of each insert and cells were allowed to invade for 21 h. Cells that did not invade were removed using a cotton swab. Invading cells attached to the bottom of the transwell were fixed with 4% of paraformaldehyde and washed once with Dulbeco’s Phosphate Buffered Saline. Cells were stained with crystal violet solution (0.1 g in 100 ml of H_2_O) and imaged in 4–6 random fields at 10× magnification using a Leica CTR 6000 Microscope (Leica Microsystems, Wetzlar, Germany) and ImagePro 6.2 software (Media Cybernetics, Bethesda, MD). Cells were manually counted from these images and used to calculate the average number of cells in each field.

### RNA isolation and TaqMan real-time PCR studies

*mRNA expression*: Total RNA was extracted from human cell lines using the EZNA RNA isolation kit according to the manufacturer protocol (Omega Bio-Tek, Norcross, GA, USA). Quantitative real-time PCR analysis was carried as previously described using the Applied Biosystem 7900HT or QuantStudio 7 Flex Real-Time PCR Systems and Assays on Demand^TM^ (AOD) specific for the genes of interest and normalized to endogenous GAPDH (Life Technologies, Carlsbad City, CA, USA)^14,58^. AODs used were as follows: GAPDH (4325792), Rac1 (Hs01902432_s1), PKCγ (Hs00177010), and pan-p63 (Hs00978340_ml). q-PCR reactions for each specific gene were run in triplicate.

*miRNA expression*: Total RNA was extracted from human cells using the EZNA RNA isolation kit according to the manufacturer’s protocol (Omega Bio-Tek, Norcross, GA, USA). A TaqMan MicroRNA reverse transcription kit (Life Technologies, Carlsband, CA, USA) was used to synthesize cDNA from 10 ng total RNA using primers specific to hsa-miR-320a (RT:002277) or RNU-48 (RT:001006) as per the manufacturer’s protocol. qRT-PCR was performed using the Applied Biosystem 7900HT or QuantStudio 7 Flex Real-Time PCR Systems using TaqMan 2× universal master mix and miRNA specific AODs. AODs used were hsa-miR-320a (TM:002277) normalized to RNU-48 (TM:001006). q-PCR reactions for each specific gene were run in triplicate.

### In silico analysis of ΔNp63α isoform, PKCγ, and miR-320a expression among the TCGA Pan-Cancer dataset

Clinical/follow-up data and pre-processed *PRKCG* and *miR-320a* expression levels among 8885 primary cancer cases derived from 32 tumor locations were obtained from the GDC Pan-Cancer TCGA dataset at UCSC Xena browser (https://xenabrowser.net/). Similarly, the ΔNp63α isoform expression levels (ENST00000354600.9 TOIL RSEM tpm profile) were downloaded from the UCSC Toil RNAseq Recompute TCGA-Pancancer dataset at UCSC Xena browser. Univariate and bivariate (Pearson’s Correlation test) expression analysis among cases were performed with the R software.

To further explore the prognostic value of the gene expression signature composed by *ΔNp63α*, *PRKCG*, and *miR-320a* in squamous carcinomas, we evaluated the CESC dataset. Briefly, a group of 296 patients with CESC and follow-up data were divided into two subgroups (Low *ΔNp63α*/Low *miR-320a*/High *PRKCG* and High *ΔNp63α*/High *miR-320a*/Low PRKCG). Discretization of the *ΔNp63α*, *PRKCG* and *miR-320a* gene expression data into low or high expression levels was performed according the StepMiner one-step algorithm of their respective profiles (http://genedesk.ucsd.edu/home/public/StepMiner/). These groups were then compared based on the overall survival (Kaplan–Meier curves and log-rank test) using the Survival R package. Fisher’s exact test was employed to compare the distribution of CESC cases with high or low expression of *ΔNp63α* and *miR-320a* transcripts.

### Statistical analysis

Experiments were done in triplicates and values are presented as the mean ± SD. Student’s *t* test was used to analyze the statistical significant changes. Significant changes (*P* ≤ 0.05) relative to controls are indicated with an asterisk.

## Supplementary information

Supplemental Figures 1 and 2

Supplemental Material
