# Supplementary figures and images for: ΔNp63α suppresses cells invasion by downregulating PKCγ/Rac1 signaling through miR-320a
Source: Cell Death Dis. 2019 Sep 12;10(9):680. doi: 10.1038/s41419-019-1921-6 (PMC6742631; doi:10.1038/s41419-019-1921-6)

Figure S1

A.

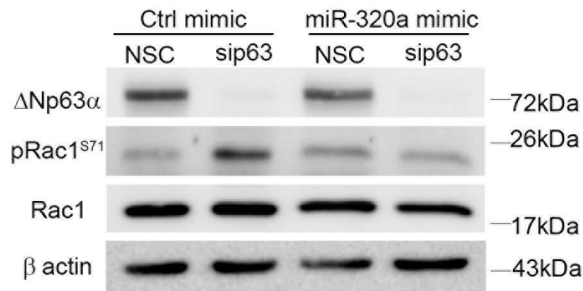

B.

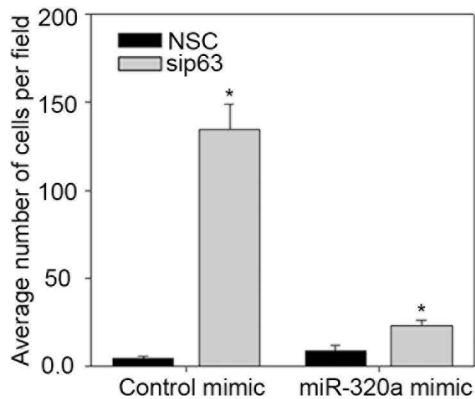

Figure S2

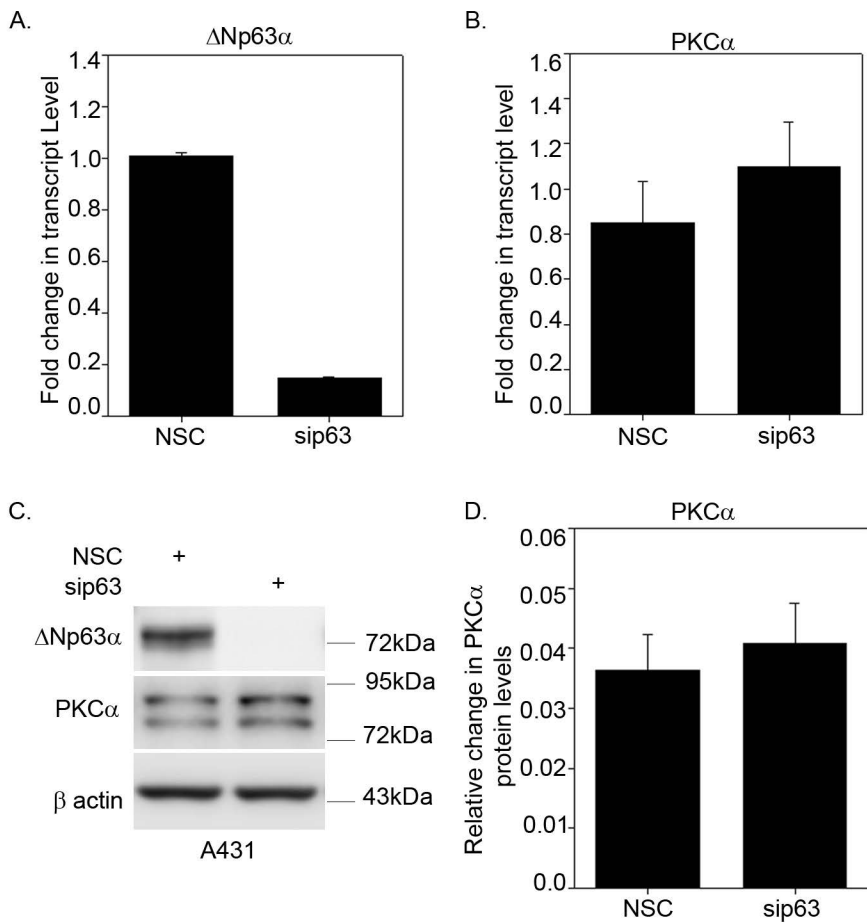

Supplement: Supplementary file 1 — Supplemental Figures 1 and 2 [file 41419_2019_1921_MOESM1_ESM.pdf]
