# Supplementary material for: ΔNp63α suppresses cells invasion by downregulating PKCγ/Rac1 signaling through miR-320a
Source: Cell Death Dis. 2019 Sep 12;10(9):680. doi: 10.1038/s41419-019-1921-6 (PMC6742631; doi:10.1038/s41419-019-1921-6)
Supplement: Supplementary file 2 — Supplemental Material [file 41419_2019_1921_MOESM2_ESM.docx]

**Supplemental material**

**Supplementary Figure 1: Overexpression of a miR-320a mimic counters the effect of ΔNp63α knockdown on Rac1 phosphorylation and cell invasion in HaCaT cells.** HaCaT cells were transfected with NSC siRNA or p63 siRNA in conjunction with a negative control mimic or miR-320a mimic for two rounds of transfections. (A) The change in indicated protein levels were measured via immunoblotting with p63, Rac1 and pRac1 (S71) antibodies as indicated. Immunoblot with β-actin was performed to confirm equivalent protein loading. At 24 hours after the second of transfection, 8.0x10^4^ cells were subjected to Matrigel-based invasion assay and the number of invading cells was quantitated after 21 hours (B). The y-axis represents the average number of cells invaded per field. Error bars represent standard deviation. Significant changes (*P* ≤ 0.05) relative to NSC controls are indicated with an asterisk.

**Supplementary Figure 2: Knockdown of p63 did significantly increase PKCα levels.** A431 cells were transfected with NSC or sip63. TaqMan based qRT-PCR was used to quantify p63 levels (A) and PKCα levels (B). Fold Changes in transcript levels of ΔNp63α and PKCα to NSC-transfected cells are shown as means ± S.E.M. from n = 6 experiments. (C) The change in indicated protein levels were measured via immunoblotting with p63 and PKCα antibodies as indicated. Immunoblot with β-actin was performed to confirm equivalent protein loading. (D) Quantification of PKCα protein bands. Relative protein values are shown as means ± S.E.M. from n = 6 experiments.
